# The Geometric Framework for Nutrition: New Insights Into How What We Eat Affects How We Age

**DOI:** 10.1093/geroni/igaa057.3100

**Published:** 2020-12-16

**Authors:** Alan Cohen, David Raubenheimer

## Abstract

The geometric framework for nutrition (GFN) is an approach to understanding the effect of nutrition considering multiple nutrients simultaneously. Originally developed in experimental studies of insects to model how nutritional needs evolve depending on ecological context, and since extended to many taxa including non-human primates in the wild, the technique is increasingly applied to understand human health and aging. Here, we invite four varied talks showcasing the flexibility and potential of this approach from the basic biology of aging to observational human studies and clinical trials. D. Raubenheimer will give an overview of the method, its history, and its applications in aging and human health. D. Wahl will present results showing how GFN can help develop diets that recapitulate caloric restriction and its effects on brain aging. S. Das will show how GFN can be used to improve the feasibility of caloric restriction in humans without compromising its effects. Finally, A. Cohen will present results showing how GFN can be deployed in an epidemiological context and used to characterize complex interactions among large numbers of nutrients in determining health. Together, these results show that a simplistic conception of nutrition as calories is far from sufficient to understand its effects on health and aging. Evolution has shaped the nutritional needs of each species for its environment, with appropriate levels of flexibility. GFN provides an approach to capture the relevant nuance, with the results presented at this symposium but scratching the surface. Nutrition Interest Group Sponsored Symposium.

